# Translating cell therapies for neurodegenerative diseases: Huntington’s disease as a model disorder

**DOI:** 10.1093/brain/awac086

**Published:** 2022-03-09

**Authors:** Anne E. Rosser, Monica E. Busse, William P. Gray, Romina Aron Badin, Anselme L. Perrier, Vicki Wheelock, Emanuele Cozzi, Unai Perpiña Martin, Cristina Salado-Manzano, Laura J. Mills, Cheney Drew, Steven A. Goldman, Josep M. Canals, Leslie M. Thompson

**Affiliations:** 1 Cardiff University Neuroscience and Mental Health Research Institute, Hadyn Ellis Building, Cardiff CF24 4HQ, UK; 2 Cardiff University Brain Repair Group, School of Biosciences, Life Sciences Building, Cardiff CF10 3AX, UK; 3 Brain Repair and Intracranial Neurotherapeutics (B.R.A.I.N.) Biomedical Research Unit, College of Biomedical and Life Sciences, Cardiff University, Cardiff CF14 4EP, UK; 4 Cardiff University Centre for Trials Research, College of Biomedical and Life Sciences Cardiff University, 4th Floor Neuadd Meirionnydd, Heath Park, Cardiff CF14 4YS, UK; 5 University Hospital of Wales Healthcare NHS Trust, Department of Neurosurgery, Cardiff CF14 4XW, UK; 6 Université Paris-Saclay, CEA, CNRS, Laboratoire des Maladies Neurodégénératives: mécanismes, thérapies, imagerie, 92265 Fontenay-aux-Roses, France; 7 Université Paris-Saclay, CEA, Molecular Imaging Research Center, 92265 Fontenay-aux-Roses, France; 8 University of California Davis, Department of Neurology, 95817 Sacramento, CA, USA; 9 Transplant Immunology Unit, Department of Cardiac, Thoracic and Vascular Sciences, Padua University Hospital—Ospedale Giustinianeo, Padova, Italy; 10 Laboratory of Stem Cells and Regenerative Medicine, Department of Biomedical Sciences, and Creatio-Production and Validation Center of Advanced Therapies, Faculty of Medicine and Health Sciences, Institute of Neurosciences, University of Barcelona, Barcelona, Spain; 11 August Pi i Sunyer Biomedical Research Institute (IDIBAPS), Barcelona, Spain; 12 Networked Biomedical Research Centre for Neurodegenerative Disorders (CIBERNED), Barcelona, Spain; 13 Centre for Translational Neuromedicine, University of Rochester, 14642 Rochester, NY, USA; 14 University of Copenhagen Faculty of Health and Medical Sciences, DK-2200 Kobenhavn, Denmark; 15 University of California Irvine, Department of Psychiatry and Human Behaviour, Department of Neurobiology and Behavior and the Sue and Bill Gross Stem Cell Center, 92697 Irvine, CA, USA

**Keywords:** cell therapy, stem cells, clinical translation, neurodegeneration, Huntington’s disease

## Abstract

There has been substantial progress in the development of regenerative medicine strategies for CNS disorders over the last decade, with progression to early clinical studies for some conditions. However, there are multiple challenges along the translational pipeline, many of which are common across diseases and pertinent to multiple donor cell types. These include defining the point at which the preclinical data are sufficiently compelling to permit progression to the first clinical studies; scaling-up, characterization, quality control and validation of the cell product; design, validation and approval of the surgical device; and operative procedures for safe and effective delivery of cell product to the brain. Furthermore, clinical trials that incorporate principles of efficient design and disease-specific outcomes are urgently needed (particularly for those undertaken in rare diseases, where relatively small cohorts are an additional limiting factor), and all processes must be adaptable in a dynamic regulatory environment.

Here we set out the challenges associated with the clinical translation of cell therapy, using Huntington’s disease as a specific example, and suggest potential strategies to address these challenges. Huntington’s disease presents a clear unmet need, but, importantly, it is an autosomal dominant condition with a readily available gene test, full genetic penetrance and a wide range of associated animal models, which together mean that it is a powerful condition in which to develop principles and test experimental therapeutics. We propose that solving these challenges in Huntington’s disease would provide a road map for many other neurological conditions. This white paper represents a consensus opinion emerging from a series of meetings of the international translational platforms Stem Cells for Huntington’s Disease and the European Huntington’s Disease Network Advanced Therapies Working Group, established to identify the challenges of cell therapy, share experience, develop guidance and highlight future directions, with the aim to expedite progress towards therapies for clinical benefit in Huntington’s disease.

## Teaser

Rosser *et al.* discuss the challenges associated with clinical translation of cell therapies, using Huntington’s disease as the primary example. They suggest strategies to address these challenges, based on the consensus view emerging from international workshops and discussion within the platform ‘Stem Cells for Huntington’s Disease’.

## Introduction

We are in an exciting phase of accelerated progress for advanced therapy medicinal products (ATMPs), which includes recent progress in stem cell therapies. The optimism around stem cell therapies is built on decades of preclinical research establishing the key principles of cellular therapies, developments in the stem cell field that are leading to a better understanding of how to generate and manufacture donor cells, and the emergence of key research methodologies in the area of genomics, epigenomics and human imaging.

Huntington’s disease is a potential indication for regenerative medicine and represents a neurodegenerative disease paradigm in which to establish principles for its safe and efficient clinical translation. Huntington’s disease is an inherited disorder which typically develops in mid-life and is characterized by progressive motor, cognitive and psychiatric impairment, seriously eroding quality of life and with a high societal impact.^1^ It is the most common monogenic neurodegenerative condition of the CNS, being caused by a CAG repeat expansion in exon 1 of the huntingtin gene. The availability of a reliable genetic test, and complete penetrance for CAG repeats above 39, mean that Huntington’s disease is reliably diagnosed in life and individuals carrying the mutation can be identified in the presymptomatic phases. These factors provide substantial power for clinical studies that seek to evaluate disease progression and/or potential modification by treatments. This, alongside the fact that Huntington’s disease features the major pathophysiological hallmarks of the most prevalent multi-genic and/or multifactorial neurodegenerative diseases and the availability of multiple cell and animal models, make it an excellent candidate in which to test, optimize and translate cell therapy, while maximizing the potential impact of addressing challenges that may cross over to other neurodegenerative conditions.^2,3^

The underpinning concept of stem cell therapy is restorative. This restorative goal can be achieved through several approaches. For example, implantation of cells that provide support for existing vulnerable host cells through a variety of mechanisms including controlled release of trophic molecules or implantation of cells designed to integrate and adopt the function of those lost to the disease process (the latter is referred to here as cell replacement therapy) are potential non-mutually exclusive approaches. Neurons degenerate throughout the brain in Huntington’s disease, but the earliest and most severe loss occurs in the striatum where medium spiny striatal neurons (MSNs), the most abundant neurons in the normal striatum, are most affected.^4,5^ Thus, one therapeutic aim is striatal neuronal replacement, with a particular emphasis on transplanting cells capable of differentiating into MSNs. Mature adult neurons will not survive transplantation so it is necessary to transplant progenitors that can differentiate into MSNs.^6^ Early studies focused on donor cells collected from the developing foetal striatum, where MSNs develop during normal development, and pilot studies in which such cells were transplanted into the striatum of individuals with Huntington’s disease have demonstrated feasibility and proven safe overall.^7^ However, collecting high quality foetal tissue and performing adequate quality control in the limited time window between collection and surgical delivery is difficult and limiting.^8,9^ This has stimulated research to derive striatal-like neurons from renewable sources such as human pluripotent stem cells (hPSCs) including induced pluripotent stem cells (hiPSCs), embryonic stem cells (hESCs) and human foetal-derived neural progenitor cells (NPCs), with initial evidence of functional improvements in preclinical models of Huntington’s disease.^10,11^ Progress is being made in establishing the mechanisms underlying improvement, for example hNPCs have been reported to differentiate into neuronal and glial populations, secrete neurotrophic factors such as BDNF, and connect with endogenous cells to re-establish neural circuitry,^11^ but further basic research to adequately address such questions continues to be essential. Furthermore, key steps towards clinical translation still require careful phenotyping of the cells being transplanted, as well as evaluating the long-term integration and behavioural outcomes of the grafted Huntington’s disease animal models.

Cellular degeneration in Huntington’s disease isn’t restricted to neurons; glia, both astrocytes and oligodendrocytes, appear to be affected from the earliest stage of Huntington’s disease and therefore glial replacement presents another exciting therapeutic avenue.^12^ Human glial progenitor cells (GPCs) are broadly migratory and can produce astrocytes as well as oligodendrocytes. Diseased astrocytes in particular appear responsible for much of the synaptic pathology in Huntington’s disease,^12–14^ and their replacement by transplanted normal GPCs has proven effective at rescuing threatened MSNs in Huntington’s disease mouse models.^13,14^ However, GPCs cannot generate lost MSNs, so it is possible that some as-yet-to-be defined combination of GPCs and either MSN progenitors or MSN-biased neural stem (or progenitor) cells (NSCs) may be optimal to accomplish the structural repair and functional rescue of the diseased striatum in Huntington’s disease. Thus, for Huntington’s disease stem cell therapy we have yet to determine the composition, developmental potency and molecular make-up of the ‘best’ donor cells.

Importantly, there is no credible evidence that non-neural cells can differentiate into neural cells, unless specifically modified (usually genetically) to do so. As such, undifferentiated, mesenchymal or other non-neural cells are not considered here as options for cell replacement therapy in Huntington’s disease, although some of the challenges considered below will nevertheless be pertinent to these cell types.

No disease-modifying treatment exists as yet for Huntington’s disease, although trials of potential therapies targeted at key pathogenic pathways are underway or on the horizon, such as various strategies to lower mutant huntingtin levels or target DNA damage repair pathway.^15^ However, these agents cannot recover cells already lost, and even decades before the motor onset of Huntington’s disease, there is measurable loss of cells in the striatum.^4,16^ Thus, cell therapy has the potential to have an important place in the treatment of Huntington’s disease for individuals with existing cell loss, especially in the absence of therapies that can be delivered in the presymptomatic stage of the natural history, and also in the event that future disease-modifiers may only slow (rather than halt) disease progression. Although we anticipate that cell therapy may be a stand-alone treatment for some patients with Huntington’s disease, graft-induced improvement could eventually be overtaken by the underlying disease process, therefore, it is important to note that cell therapy is likely to be fully compatible with other potential therapies on the horizon, thus addressing both existing and ongoing cell loss and potentially making it widely applicable. It is also possible that implanted cells could be engineered pre-transplantation to deliver disease-modifying molecules.

We propose that it is important to pursue stem cell therapies for Huntington’s disease, with the intention of meeting the need for therapeutics in Huntington’s disease and to help provide a road map for translation of cell therapies in other neurodegenerative conditions. In order to achieve this in the safest and most efficient way, we have established ourselves as an international consortium of experts, which we call ‘Stem cells for Huntington’s disease’ (SC4HD; www.sc4hd.org).^17^ SC4HD aims to provide a platform for discussion and to share experience in order to provide guidance and to generate a robust clinical development plan across a range of stem cell-based therapies for Huntington’s disease. The consortium works closely with the European Huntington’s Disease Network (EHDN) Advanced Therapies Working Group (ATWG: http://www.ehdn.org/advanced-therapies-wg/), which aims to address similar issues for both cells and molecules, and with the California Institute for Regenerative Medicine (https://www.cirm.ca.gov/), that seeks to provide stem cell-based therapies for a range of human diseases. Here we set out a consensus document that identifies key challenges to clinical translation and indicates the next steps needed in order to move forward safely and effectively to the next phase of this work.

## Challenge 1: Defining principles that can be used to guide decisions to advance a potential stem cell therapy towards a first-in-man trial

### Nature of the challenge

Criteria that indicate a high likelihood that preclinical benefit in animal models will translate to improved human disease outcomes are not yet defined for Huntington’s disease, nor for other neurodegenerative diseases. Cell therapy candidates typically emerge from a series of *in vitro* and *in vivo* basic science studies, but standardization of outcome measures and models is lacking. To some extent, the animal models used and the specific assessments required will be dependent on the therapeutic and the proposed underlying mechanism. For example, whether the therapeutic is designed to replace cells lost to the disease process, perhaps with the re-establishment of damaged circuitry, what the intended distribution of those cells is, or whether the therapeutic is designed to deliver trophic molecules or a combination of each, will guide the nature of the preclinical assessments. However, some standardization of outcomes, at least for specific therapeutic strategies, would facilitate comparisons between studies and the validation of finding.

An additional challenge is to define principles that could guide the transition from preclinical development to clinical translation; that is, the point at which the preclinical data are sufficiently compelling to consider the candidate as a serious therapeutic possibility, and to engage in potentially costly and time-consuming activities such as toxicology studies and discussions with regulators.

### Strategies to address the challenge

Defining principles that support translation to first-in-man studies will require attention to choice of preclinical models, standardization of key outcome measures, and defining principles for progressing to clinical translation. Key considerations include: (i) the numbers and types of relevant *in vivo* models and numbers required for well-powered safety and efficacy studies; (ii) the extent to which the mechanism of action of the cell product is defined; (iii) the outcome being assessed including cell fate, potency, safety, and long-term efficacy; and (iv) standardized readouts that may be relevant and predictive of outcomes in a human trial.

#### Choice of preclinical model

The choice of a given model will be guided by the goal of the study; an optimal model may be different for assessing cell fate versus one used to evaluate mechanism of action of a stem cell product. Preclinical efficacy studies to evaluate the potential efficacy of a neural cell-based therapy have typically been carried out in mouse or rat models of Huntington’s disease, both genetic and toxin models of the disease. Genetic models recapitulate aspects of human disease, including the presence of a CAG repeat expansion leading to expression of an expanded polyglutamine repeat RNA and protein, toxic mutant huntingtin species and disease progression. There is an extensive range of genetic rodent models of Huntington’s disease,^18,19^ including rapidly progressing transgenic mutant huntingtin fragment models and slower progressing full length transgenic and ‘knock-in’ models. Although most genetic models are currently in mouse, rat models exist and are becoming better characterized and large animal models (e.g. pig, sheep, non-human primate) are in various stages of development and use as described below, albeit primarily utilized in later stages of cell therapy development. Toxin models in both mice and rats may be utilized to evaluate specific questions involving placement, migration of stem cell products within a damaged niche, and integration into neural circuitry, that cannot always be addressed in genetic models that to date present relatively little cell loss.^20^ Thus, thorough testing of a cell product may require use of more than one animal model and a framework to guide selection of animal models for cell therapy studies are needed.

#### Standardized outcomes

Efficacy testing is an essential component of preclinical studies, but it is challenging to define the most relevant outcome criteria, given the current lack of validated therapies that have moved from preclinical studies to disease modification in human patients. Typically, behavioural assays have been used to assess efficacy in Huntington’s disease mouse models,^3,20^ however we need to understand more about the relationship of any given assay or measurement to changes in human disease and most relevant translational end points. Restoration of molecular and cellular phenotypes altered in Huntington’s disease models and in human disease including gene and protein expression, protein homeostasis, trophic factor activity, electrophysiology to reflect circuitry, and neuropathological improvement may be highly informative as potential end points and may be more readily standardized and related to human disease. Developing a better understanding of how individual measures relate to human disease and suggesting core outcome sets may be useful, although rather than adopting a single primary outcome and specifying secondary outcomes, as is typical in human clinical trials, it could be argued that a diverse array of assays is needed, including those that test the proposed mechanism of action, in order to maximally inform clinical progression. Finally, there are a range of other technical considerations such as using immunosuppressive drugs for human xenografts versus using immunocompromised mice to alleviate rejection of a given cell product.^21^

#### Defining principles that could guide clinical translation

Establishing principles that aid decisions to progress a cell product to clinical translation will need to take account of a range of cell products and purposes and will need to be based on expert consensus through leveraging the experiences across multiple disciplines. Confidence in decisions to progress to clinical translation would be increased by testing in more than one laboratory, which will in turn be dependent on standardizing outcome measures as discussed above and by compiling data in standardized formats.

## Challenge 2: Cell manufacturing, scale-up, safety and compliance of cell product for human application

### Nature of challenge

Once a cell-based candidate is identified, early safety testing of cells is essential and may include assessing the potential for tumour formation, neural overgrowth of immature neural progenitors,^22,23^ and unwanted/uncontrolled cell migration. Toxicology and tumorigenicity studies are usually undertaken at least in rodents and require good laboratory practice (GLP) services, but the lack of clear standards for toxicity testing and a need to agree these with the regulators for individual applications is a challenge.

There are further challenges related to the cell manufacturing process. As ATMPs, cells must be produced in compliance with good manufacturing practice (GMP), which is primarily designed to ensure safety of the cell product. GMP involves design of quality control systems to ensure compliance of the product's quality and safety attributes with previously defined specifications. There are clear quality standards in place in the EU and USA for donation and harvesting, testing, processing, preservation, storage, and distribution of human tissues and cells^24,25^ and specific EU GMP guidelines for ATMP manufacturing came into force in May 2018 (Part IV-GMP requirements for Advanced Therapy Medicinal Products),^26,27^ which detail the requirements for manufacture of cell products under GMP conditions, including requirements for the personnel participating, facilities, equipment and quality control, among others. Although the requirements of the GMP process are well-defined, achieving them presents a number of challenges.

A key challenge is to define the target product profile (TPP) which will guide the steps of GMP translation. The first step in generating a cell product for clinical use is to translate basic research procedures to a GMP quality system, which entails producing a documentary system for managing the manufacturing process, quality control and quality assurance of ATMPs to ensure high quality standards. Achieving this requires initial application of a risk-based approach, according to the GMP standards to evaluate the whole procedure and to detect points of high risk that need a mitigation/control plan. The next step is validation, to ensure robustness, homogeneity and quality of the manufacturing procedures. This includes training and qualification of manufacturing personnel in any GMP procedure by carrying out media fill or aseptic process simulation (APS), and use of those specific procedures to test reagents, starting materials, in process control (IPCs) and final specifications. None of these steps can be achieved without clear and detailed specification of the TPP, which in turn depends on the purpose of the cell therapy, for example whether the aim is to replace specific neuronal and/or glial populations, and needs to be worked out on a case-by-case basis.

There are further challenges in establishing an optimal cell manufacturing process and in accommodating further refinements to these processes following lock down of the protocol. In this sense, it is equally important to control every stage of the manufacturing processes. As cell cultures are living systems, controlling intrinsic variability in cell growth or cell differentiation, among other critical aspects, between batches or donors is a challenge that researchers encounter. For this reason, it is important to set up a sampling plan based on IPCs. Other challenges include scale-up to expand stem cell populations and cell banking, before differentiating the cells to a specific cell population, and adapting procedures and equipment to large scales batches. A risk-based approach might be useful to plan appropriate manufacturing scale-up stages, since basic research procedures may not be able to generate the large numbers of cells necessary for human therapeutic application.

### Strategy to address the challenge

#### Standardizing safety testing

Currently, regulations vary, and in some countries, toxicology and tumorigenicity studies must be done in at least two different species, with rodents being the first option. Furthermore, while proof-of-concept studies are done in Huntington’s disease animal models, toxicology and tumorigenesis studies may be carried out in control animals if local medical agencies accept it. Development of standards for toxicity testing would be valuable and could include issues relating to design of studies such as whether to include spiking studies to evaluate tumour formation, as well as management of the study, oversight and training. Such standards could be usefully constructed across a number of neurological diseases and can be informed by outcome data emerging from ongoing clinical trials.

#### Target product profile specification

As outlined above, an important challenge is to clearly define the TPP. Although some flexibility at the early clinical stages (phase I/II) is accepted, it is necessary to specify the minimal criteria that define the products in terms of safety and effectiveness, which includes establishing the quality attributes of the final product such as cell number, dose, cell phenotype and karyotype among others. Product specification will, of course, depend on the specific cell therapy approach. For example, if the purpose is to substitute the degenerated MSNs, the cellular features of the transplantable MSN-committed neuronal progenitor cell must be defined, and this may require validation in animal models unless reliable surrogate markers of a successful transplant can be established. Alternatively, if the purpose is to perform *ex vivo* gene therapy using cells to release protective factors, the released dose of the factors may be more important than the specific features of the cells. Although TPP specification depends on the specific aims of the product, principles for determining the key elements of the TPP could usefully be established and will be an aim for SC4HD moving forward. For example, principles could be established to guide the process of determining the efficacy, which are likely to align, at least to some extent, with the requirements for progression to clinical trial as discussed above.

#### Control of manufacturing processes

Since cell cultures are living systems, it is crucial to control all stages of the production process, such as cell expansion or cell differentiation. In addition, given that the aseptic processes for obtaining cellular products are complex and can take even weeks, it is essential to establish a sampling plan that allows guaranteeing correct dynamics of the culture. In this sense, IPCs or in process testing should as well be planned according to the complexity of the procedure. Both are crucial to understand the dynamics of the cell culture as well as the critical points of the procedure. In process testing should occur when critical further steps in the manufacturing process are taken, such as additional scale up, to allow manufacturing halt or shutdown if the IPC reveals a problem. For this reason, a sound knowledge of the production process is required, not only in the regulatory frame but also in the biological knowledge of the product (cell growth, morphology, doubling times, proliferation rates, cell type markers and quantitative criteria and standards for these markers). For example, morphological observations during stem cell differentiation such as rosette formation during MSN differentiation could be a necessary IPC that guarantee the correct differentiation procedures.^28^ Establishing the analysis of the presence of key factors during neuronal or astrocytic differentiation could indicate minimal Go/NoGo percentages of cell differentiation at relevant stages. Establishment of biological product assays and comparisons of cell products will be helpful for clinical development.

#### Scaling and stability

The clinical application of stem cell derivatives usually requires scale-up. This stage may involve the incorporation of 2D cell expansion systems with large surfaces such as cell factories and, if the cell cultures are carried out in suspension, cell culture systems for large volumes. However, sometimes, and due to the large number of cells required per patient, bioreactors or other automatic cell expansion systems can be more advantageous. In addition, generating and characterizing a Master Cell Bank and subsequently a Working Cell Bank of hPSCs, which will be used as starting material, should be considered before moving towards the manufacturing step. It is also highly recommended to cryopreserve the final product, for example neuronal progenitors committed to an MSN or glial phenotype, in ‘Drug Substance Banks’, which should be fully characterized before implanting into participants, although cryopreservation at this stage may not always be possible. When generating a final product bank that is ready to be grafted is not possible, for example if mature MSNs cannot be banked, the exact procedure to generate the final product must be defined. Cell manufacturing should be aligned with the clinical trial approach and the clinical requirements that the cellular product must fulfil. Clinicians and researchers can work closely to define and design the whole process in order to address all challenges mentioned above to obtain high quality and effective products.

Although differentiation protocols will be specific for the target cell type, many of the related challenges in translating these to GMP standards are disease and cell type agnostic and applicable to neurodegenerative diseases other than Huntington’s disease. Significant progress in addressing many of these challenges has been made over the last decade for manufacture of hPSC-derived dopaminergic projection neurons for Parkinson’s disease by member labs of the G-Force consortium (an international collaboration for cell transplantation in Parkinson’s disease: http://www.gforce-pd.com) and associated biotech companies.^29^ In contrast to Parkinson’s disease, where specification of the graft product (dopaminergic neurons) is common across most major players in the field and the number of cells required is relatively small, a much wider variety of neuronal and glial donor cells are currently being considered for cell therapies in Huntington’s disease.

## Challenge 3: When to consider the use of large animal models

### Nature of challenge

An important question for preclinical safety and efficacy studies relates to when and for what purpose large animal studies should be incorporated. Key potential advantages of large animals are greater brain volume than rodents and anatomical and functional organization closer to that of humans. For cell therapy, the dose of biological product required to obtain a functional effect is best modelled in a brain more comparable to the human brain, given the numeric scale-up of surviving cells to deliver a clinically relevant response and the longer distances required for innervation and circuit restoration. Moreover, the use of large animals permits testing of delivery devices and/or techniques intended for use in human subjects along with the therapeutic product, which should increase the predictivity of results. There are several species that can be used to assess these parameters, in either control animals or Huntington’s disease models, depending on the specific question to be addressed. A number of large animal genetic models of Huntington’s disease, obtained through transgenesis or viral overexpression, are available or in development, including pig, sheep and non-human primate models.^30–32^ As regulatory agencies do not strictly require either safety or efficacy testing in large animals, consideration of the circumstances in which these studies would be either necessary or highly valuable would be helpful.

### Strategy to address the challenge

At a minimum, large animals offer the ability to assess five critical parameters, albeit usually in a relatively small number of animals, before taking a cell therapy to the clinic: delivery route, device testing, the survival of cells, their biodistribution, and the safety of the approach. The value of using large animal models, Huntington’s disease or controls, centres around the functional (with respect to behavioural and imaging outcomes) and adaptive immunological perspectives that can be used to assess the longer-term survival and biodistribution of cell therapy products in a context that is closer to humans than rodents. Key drawbacks of large animal models are their cost, in some cases their generation time (transgenics), longer latency to study the effect of cell therapies due to longer time required to generate the models and for implanted cells to mature and ethical views on their use.

Overall, the specific question drives the choice of model to be used, whether to use healthy or a disease model, and if using a disease model, which of the available ones. For example, although generating inflammatory lesions or huntingtin overexpression only mimics certain features of the disease in humans, this might be pertinent to address specific questions such as blood–brain barrier permeability, rejection mechanisms, or the effect of neuroinflammation on cell survival.

The follow up techniques used to characterize the safety and viability of the cells are critical in terms of predicting clinical outcome. As such, the possibility to selectively study motor and cognitive behaviour, and potentially link graft size and placement within the caudate and putamen to the measured outcomes, illustrates the preclinical pertinence relevance of primate models compared to rodents. Imaging tools such as PET and MRI can be linked to specific anatomical and functional regions in a large animal brain in a way that is not achievable in the smaller rodent brain, and can be advantageous when assessing the functional impact of axonal outgrowth from the grafted cells and their connectivity to target regions that are spatially remote in large animals compared to rodent brains. For example, MRI has recently been used, not only to determine graft placement and volume as in rodents, but also to longitudinally monitor adverse effects such as inflammation, oedema or haemorrhage after cell transplants undergoing rejection thanks to the higher anatomical resolution achieved when imaging a large primate brain and the similarity of the immune system to that of humans.^32^ The role of the blood–brain barrier and the local reaction of the immune system to cellular grafts can be explored, to reduce the risk of rejection in patients and improve cell survival and differentiation, both of which will impact on the efficacy of the therapeutic strategy. Another issue that may be more satisfactorily addressed in large animals than rodents is the effect of long-term training of a graft on Huntington’s disease-specific cognitive features, such as perseveration, that are difficult to assess in rodents. However, when considering the use of human cells in animals, long-term immunosuppression is required to prevent rejection of the xenotransplant, which might be challenging in practice and costly. The use of animal species that have an immune system similar to that of humans, such as non-human primates, or rodents with a humanized immune system, may also be considered.

Another advantage of large animal models is the volume of biological fluids, such as blood and CSF, that can be collected longitudinally to follow up adverse events or investigate validated progression markers and disease modifying markers.^21,33,34^ The availability of large quantities of post-mortem tissue from animals transplanted with cell therapy products allows application of various biochemical and molecular biology techniques as well as standard immunohistochemistry in the same individual, and permits linkage of these results to the *in vivo* functional outcomes, thus providing an invaluable source of data to establish the consequences of therapeutic interventions and to inform the design of clinical trials. Finally, dosing studies may be desirable to support selection of the initial human dose, although this should be regarded as a guide dose and does not preclude the need for human dosing studies which will explore the effects of the treatment in humans at lower doses.

In summary, the use of large animal models and in particular non-human primates has ethical, practical and cost-related issues that need to be considered on the basis of the question to be addressed. All the issues outlined here are complex and weighing up the pros and cons requires more detailed consideration in order to provide guidance for researchers interested in the use of large animal models for the translation of preclinical cell therapy strategies to the clinic.

## Challenge 4: How can we optimally deliver cells to the brain?

### Nature of the challenge

The impermeable nature of the intact blood–brain barrier means that systemic cell delivery is not effective, and while barrier breakdown in certain conditions affords the possibility of small molecule access, the inability to spatially constrict and or deliver to distant impermeable areas, means this strategy has likely limited applicability to cell therapy at the moment. In addition, the specific brain area in which the cells are transplanted may also play a crucial role in the graft survival, integration and functionality of the graft, as well as on the immune response generated upon transplantation.^35^

The development of optimized devices has lagged behind that of the cell therapies^36^ for reasons of research funding and regulatory confines (*vide infra*), with clinical trials using in-house manufactured devices or off-label use of commercial catheters designed for gene therapy delivery. Despite the well established principles of safe stereotactic neurosurgery for functional stimulation and ablation, efforts at simple scale-up of delivery devices from rodent to human brain have not met with unqualified success,^9,37^ the main issues being the need to deliver significantly greater numbers of cells over a larger volume of brain, using delivery devices that scale poorly. Studies have shown significant issues with cell sedimentation within the delivery catheter^38^ back reflux of cell therapy product along the delivery needle tract leading to ectopic delivery and engraftment failure^9^ and poor survival.^39,40^ Moreover, optimal targeting of cell delivery remains largely unexplored. Striatal cell loss in Huntington’s disease is not uniform, progresses over time and is associated with neuroinflammation.^41^ Therefore, whether to deliver the cell therapy to areas of maximal cell loss or cell preservation or with greater or lesser levels of neuroinflammation remains unknown, as imaging these variables remains experimental.^42^ Additional challenges to be addressed include reducing the number and length of delivery tracks, developing technical expertise, intervention fidelity, efficacy assessment and regulatory considerations.

### Strategies to address the challenge

#### Optimizing device design

All clinical studies of cell therapy in Huntington’s disease to date have used simple needle/cannula devices, requiring multiple cortical penetrations to deliver cells into a co-axial preformed track, mostly via an end aperture. While it is not possible to directly assess the early performance of cell delivery in these trials, there was a high degree of graft failure in many on subsequent imaging. Animal studies have shown high rates of donor cell death immediately after implantation with these simple catheter designs,^39^ and this is likely to be a significant contributor to poor engraftment because of early cell loss due to hypoxia within the bolus of delivered cells. In the small number of cases from clinical trials examined at post-mortem, ectopic graft tissue, presumably from cell reflux, was also associated with a poor outcome.^9^ Large animal models (sheep, pig and non-human primate) are a requisite for evaluating *in vivo* delivery performance, as the biophysical parameters constraining cell delivery are very different in small animal brains compared to human brain, both in physical dimension and the effects of disrupted anatomy caused by disease e.g. enlarged perivascular spaces in the brain in Huntington’s disease brain.^43^ While stepped designs at the distal catheter end for convection enhanced delivery of gene therapies have reduced therapy reflux,^44^ this has not yet been evaluated for cell delivery, but may hold some promise. The significant cell sedimentation occurring within the delivery device over the long delivery times needed to optimize cell survival also leads to non-uniform product deposition as well as significant reflux.^38,45^ This may be partially mitigated by suspending the cells in delivery gels rather than in liquid solution, although this adds further regulatory complexity for toxicology.

#### Optimizing delivery protocols

Cell therapies need to be delivered within a fluid medium and while a delivery rate of 5–10 µl/min has been considered optimal,^46^ recent bio-mechanical studies have shown surprising effects on cell differentiation depending on the needle tip diameter and delivery rate,^47^ revealing further complexities to address beyond cell viability.

Strategies to improve the distribution of delivered cells have utilized side apertures in the delivery cannula in either a static fashion with simultaneous delivery over a defined length of the distal cannula,^46^ or single level apertures that can be rotated to deploy grafts in a 3D distribution as the cannula device is withdrawn,^48^ the latter showing long-term graft survival in Parkinson’s disease patients.^49^ Strategies utilizing novel radially delivered catheters with manoeuvrable tips are being developed in order to minimize the number of major needle tracks required^50^ whilst allowing cell delivery to a greater brain target volume. Early work showing that delivery cannula size affected graft viability^51^ has led to the development of microcannulas for cell delivery^52^ which in combination with radial delivery appears to show superior graft dispersion and less cell reflux in large animal models.^53^ While promising strategies, all these devices remain experimental for the moment, which raises issues around device regulation (*vide infra*).

#### Surgical expertise

Whatever the technical details, surgical interventions of this nature are time consuming, expensive and require expert centres where such interventions can be delivered. An important challenge that needs to be addressed to maximize clinical trial utility and future trial scale-up is that of surgical intervention fidelity (i.e. that the procedure is standardized so that product delivery and distribution is reproducible and as consistent as possible).

#### Assessing device related outcomes for clinical efficacy and regulatory approvals

Further challenges arise in efficacy assessment across both regulatory and clinical outcome domains, where it is of primary importance to discriminate between the performances of the device and the therapies it delivers. These are logically sequential and interdependent (e.g. accurate delivery and distribution of a cell therapy, early cell survival/integration and subsequent detection of a clinical effect). Currently we do not have established protocols for accurate and non-invasive clinical imaging of very early cell delivery and survival, and so efficacy can only be inferred indirectly from the success of the resulting therapy, as opposed to its specific delivery. This is especially problematic in neurodegenerative diseases where clinical benefit of cell transplantation may only be seen in the altering of disease progression over relatively long periods of time. Consequently, the early failure of a delivery device is therefore invisible to the later assessment of graft efficacy.

The consequent regulatory implications of this interdependence have neither been clarified nor addressed adequately. Indeed, the different approaches taken by the various regulatory agencies—the Food and Drug Administration, and European Medicines Agency, and the UK the Medicines and Healthcare Products Regulatory Agency—for the approval of ATMPs and their delivery devices, further complicates international comparison, evaluation and regulation. This in turn discourages iterative device development with manufacturers, and potentially creates a market of monopoly where companies invested in ATMPs could control the market for devices and stifle the development of devices not linked to their ATMP. One of the aims of the recently formed EHDN Surgical Delivery Task force is to provide specific guidance from clinical researchers to regulatory agencies on these issues.

## Challenge 5: Designing clinical trials in practice

### Nature of the challenge

Subject to appropriate regulatory approvals being in place, including those in relation to the product and the device, the first human studies will typically focus on safety before transitioning to exploratory therapeutic trials of relatively short duration in well-defined relatively homogeneous patient populations. It is common in these situations to include surrogate end points and, where relevant, to consider single arm designs in which all participants receive the experimental treatment with the objective being to establish proof of principle that warrants further investigation in a later definitive trial. Traditional phase I dose escalation studies that measure maximum drug toxicity are likely to be difficult to apply to the evaluation of such targeted therapies, given that cell therapies are not reversible and possible adverse outcomes could include graft overgrowth/tumorigenesis that could take months to become apparent. However, it will be important to establish the optimal dose, possibly through sequential cohort evaluations.

There are several constraints associated with undertaking novel experimental surgical interventions and cell therapies. The disease targeted for treatment and the route of administration are highly influential in determining the trial design of choice. The fact that cells are being delivered via a surgical approach into the brain places both ethical and practical constraints on the numbers that can/should be included in the first study. The relative rarity of Huntington’s disease and the importance of minimizing bias in these early-stage evaluations, for example relying on the use of quantitative assessments in open label trials, must be acknowledged and considered in the planning of early phase trials while recognizing that consensus on core outcome sets, namely a standardized set of outcomes that should be measured and reported as a minimum, is urgently needed. In cell therapies these will at a minimum extend from assessment of graft function to that of clinical disease status and functioning in daily life.

### Strategies to address the challenge

#### Achieving efficient clinical trials whilst conforming to regulatory standards

In rare diseases, implementing less stringent criteria (for example the use of one-sided testing or changing the type I error rate) in outcome evaluation may be worth considering. In this respect, it will be important to undertake consensus work involving the Huntington’s disease community (professionals and patients) and regulatory agencies to define acceptable levels of evidence that justify progression to definitive evaluation, and to determine which objective end points can be used to guide decision making.^54^

In small sample sizes, randomization will not always achieve its goal of balancing characteristics between treatment groups and therefore it is important to consider alternatives to the typical randomized parallel group design and to explore plausible trial designs that will minimize total sample size requirements and/or reduce variability/heterogeneity.^55,56^ This may, for example, include the use of repeated measurement outcomes in within-patient designs or trials within cohorts. It is however important, when considering the use of historical control data or observational data from disease registries, that methods to account for confounders are also taken into account.^57,58^

Given the very early stage of development of these novel therapeutics in a rare disease such as Huntington’s disease, it may be important to start by focusing on single arm early phase designs with an initial focus on graft survival and growth, and on safety and acceptability of the intervention as a whole, before moving to the evaluation of efficacy in phase III trials. Even when moving to efficacy evaluation, it will be critical to consider multiple design factors such as patient numbers, appropriate control groups, and whether there is any clear rationale for placebo surgery.

#### Placebo controls

The use of placebo-controlled designs is an important component in the rigorous evaluation of new therapies, both to account for the patient’s expectation of effectiveness, and to establish any neurobiological effects of the intervention.^59^ It is however important that placebo interventions be minimally invasive and associated with as little risk as possible. The importance of controlling for placebo effects is particularly relevant when outcome assessment is reliant on patient-reported measures. Thus, when therapeutic outcomes (for example with the use of digital sensors or computer-based assessments^60^) can be objectively quantified, and valid and reliable surrogate measures of efficacy defined, it may not always be necessary to account for the psychological placebo effect.^59^

Whilst the availability of placebo control data is highly relevant in terms of evaluating safety, particularly in the immediate postoperative period, in complex surgical interventions, the associated surgical risk of placebo must be considered. While some compromises as to the invasiveness of surgical placebos may be entertained, such as scalp incision and partial burr hole rather than dural penetrant cannulation, the larger issue is whether any such surgical placebo interventions remain reasonable in the current era of mechanistically based surrogate outcome measures and large-scale natural history studies. More broadly then, as a community we need to consider whether such alternative information can allow the development of trial designs sufficient to establish treatment efficacy and specificity thereof without defined surgical placebo.

In those cases where surrogate outcome measures are not be available or validated, and a placebo procedure, of whatever level of complexity, is undertaken, it is important to ensure that sufficient time is allowed for comparison of active and placebo arms before placebo participants are offered entry to the treatment arm. For example, for cell therapeutics intended to functionally integrate into extant neural circuits, therapeutic efficacy might take months to become apparent and years to become optimal.^61^ The time course over which efficacy develops may even be so long as to prevent the treatment of patients initially assigned as placebo controls. In these instances, as well as in rare or rapidly lethal disorders for which patient recruitment may be too difficult to enable the effective recruitment of placebo groups, large-scale natural history studies may already provide sufficient data as to the likely clinical course of well-defined patients and could obviate the need for matched placebo controls. In the specific case of Huntington’s disease, large population prospective studies such as TRACK-HD and ENROLL-HD may provide enough information as to the natural history and course of Huntington’s disease so as to constitute an even more accurate control comparator than that of concurrent placebo controls which, however well matched, may comprise a much smaller, more variable, and potentially less representative sample, than that afforded by population-based natural history studies.

## Challenge 6: Developing a framework for patient selection and follow-up in cell therapies studies

### Nature of the challenge

Patient selection and identifying batteries of suitable, sensitive outcome measures that don’t overburden participants are critical trial design issues for all neurological conditions. Despite the monogenetic nature of Huntington’s disease, between-subject variability exists in disease onset and progression of Huntington’s disease, with heterogeneity of presentation and rate of disease onset and progression attributed to genetic and lifestyle factors,^62,63^ creating challenges in designing robust clinical trials. Such challenges become more pressing for trials of complex therapies, such as cell therapy, due to additional constraints.^64^ Unlike reversible, more rapidly acting pharmaceutical agents, complex therapies involving a neurosurgical procedure are likely to involve a series of small iterative studies for a prolonged period during development of the therapeutic, placing a special emphasis on the need for sensitive, objective, outcome measures. Another consideration is that the minimum follow-up time to allow the graft to mature to the point of exhibiting functional signs that can be attributable to grafted connections can be long. For example, for MSN replacement this is estimated as 12–24 months,^65^ but certainly does not reach asymptote until 10–14 months post-transplantation,^65^ and in previous Huntington’s disease cell therapy trials, improvement was detected at 18 months and gradually increased until 4 years post-transplantation.^66^ This is significantly longer than the equivalent allograft in rodents where maturation has been reported as being little as 3 weeks post-transplantation and highlights the need for long-term follow up in trials of cell transplantation.

### Strategy to address the challenge

#### Patient selection

Patient selection and the choice of primary and exploratory outcome measures need to take account of phenotypic variability (with consideration given to narrowing the age range and disease stage of recruitment to reduce phenotypic variability), the stage of trial, and should reflect what is known about the mechanisms of the therapeutic candidate; for example, therapeutic products that increase levels of neurotrophic factors in the striatum may also rescue cortical grey matter loss. They should be modified by ongoing knowledge and a better understanding of the pathogenic mechanisms. For example, it is known that instability of the CAG repeat region in post-mitotic brain tissue is a key cause of phenotypic variability in Huntington’s disease and that this is driven by identifiable factors, such as genetic variation in proteins involved in the DNA repair process.^15^ It may be possible in the future to use this information to predict progression trajectories more accurately, and thus to use this information to design trial enrichment strategies. Cell therapy trial subjects should probably be at an early stage of the disease process for safety of delivery, the risk of postoperative parenchymal or subdural haemorrhage having been noted in previous Huntington’s disease cell therapy trials,^9,67^ for subjects’ ability to understand and participate in the scheduled assessments, and considering the need for prolonged postoperative assessment in order to assess efficacy. Criteria to reduce the risk of alloimmunization (such as prior exposure to stem products or blood transfusion) should also be considered.

#### Safety monitoring

Previous studies, the largest in Huntington’s disease being the Multi-Centre Intracerebral Graft in Huntington’s disease (MIG-HD) trial,^9^ largely based on use of CAPIT-HD^68^ provide a starting point for designing both safety and longer term assessment and emphasize the importance of baseline and serial studies including early and later timepoints. However, future studies may consider including new objective digital assessments to improve reproducibility and frequent measures in small cohort of patients (e.g. Lunven *et al*.^69^). Safety assessments for early-phase cell therapy trial in Huntington’s disease have included MRI to assess targeting accuracy and monitoring for signs of local or diffuse inflammatory response or rejection. CSF analysis can be used to detect signs of CNS inflammatory responses and laboratory studies should include human leucocyte antigen (HLA) antibodies, and potentially other markers of inflammation to assess risks of rejection. Biofluid biomarkers have also been developed, including CSF mutant huntingtin protein levels and plasma neurofilament light protein,^34,70^ although the timing of biomarker sampling should take account of the likely impact of temporary blood–brain barrier disruption during and immediately after neurosurgical implantation.^71^ Supplementing clinical assessments with validated quantitative assessments designed to minimise the potential for rater influence in outcome assessment should also be considered.^72,73^

#### The importance of long-term follow-up to measure repair beyond replacement

In Huntington’s disease transplantation trials to date, foetal ganglionic eminence (from which the striatum develops) has been transplanted into the striatum to replace degenerating MSNs, with the expectation of re-establishing degenerated anatomical circuitry over time. Typically, participants returned 6–12 months post-surgery to be assessed on a wide range of outcome measures including neuroimaging^66,67,74,75^ in order to evaluate early functional improvements as an indicator of graft integration and circuit reconstruction. Neuroinflammatory biomarkers obtained from circulating fluids such as blood or CSF have been included in several studies to analyse parameters such as donor-specific HLA antibodies to monitor the immune response,^76^ interleukins such as IL4, IL6 andIL10, or C-reactive protein to assay inflammation in a minimally invasive way.^77,78^ Neuroinflammatory biomarkers can also contribute to monitoring of the immune response following engraftment, and thus be utilized to shape the most adequate regime of immunosuppressants on an individual participant basis. The development of new biomarkers to assess both inflammatory responses to the graft the chronic neuroinflammation occurring in Huntington’s disease would be highly valuable. Importantly, after the initial more intensive assessments, participants will require long-term follow-up (perhaps even for life) to reassure the clinical and scientific community of the longer-term safety of the grafted material. As mentioned above, a disease registry [for example Enroll-HD (https://enroll-hd.org/), a worldwide observational study for Huntington’s disease families], may be utilized for the purposes of long-term follow up. Using registry follow-up data not only reduces the burden of visit attendance on the patient but also ensures high quality data and ongoing safety monitoring. Finally, increased levels of participant physical and mental activity and specific training may modify graft morphology and circuit reconstruction, leading to an understanding that training may be important for optimal graft integration.^79,80^ Thus, it is also likely that enhancing general activity, engaging in directed aerobic exercise, and task-specific training will be important components in any effective post-surgical transplant rehabilitation programme. Indeed, given clear evidence of the role of environmental enrichment in preclinical populations it is somewhat surprising that there has been as yet, little attempt to evaluate a potential assessment of life-style factors which are likely important co-variates to include in future evaluations.

## Challenge 7: Post-transplantation management to maximize graft survival and integration/immunosuppression

### Nature of the challenge

Management of the immunogenicity of the graft and of the host’s immune response to it is a major challenge. The relative immune privilege of the brain led to many neural transplant studies to date taking an approach whereby immunosuppressant therapy is administered over the period during which the blood brain barrier is disrupted (that is, following brain penetration and delivery of cells) and then withdrawn. Arbitrarily, this has translated to immunosuppressant administration being maintained for a period of 6–12 months, although in some studies none was given. There is post-mortem evidence that grafts survive many years after immunotherapy withdrawal,^7^ but there is also some evidence of allogeneic graft rejection due to alloimmunization to foetal donor antigens^81^ and some post-mortem evidence of increased inflammatory reaction around grafts^82^ suggesting that careful consideration of the need for immune suppression and duration of treatment is necessary.

### Strategies to address the challenge

Tackling graft–host interactions is the only way to ensure the long-term survival of cell therapy grafts and thus ensure their long-term therapeutic activity. Oral immunosuppression of recipients is the current standard option to manage graft immunogenicity, despite imposing increased risk of cancer, infections and cardiovascular diseases when given long-term. Post-transplant immunosuppression regimes vary. Early pilot studies using allogeneic foetal ganglionic eminence as the donor tissue opted for CyA treatment, used either alone,^74,83,84^ combined with prednisolone^85,86^ or as a component in triple immunotherapy.^75,87^ When analysing the administration of immunosuppressants in several clinical studies using foetal cell grafts in Huntington’s disease, the major benefits for graft survival seem to be associated with the use of triple immunosuppression (CyA, azathioprine and prednisolone). However, associated adverse effects of immunosuppressants have to be strictly monitored and rapidly addressed by the supervising clinical team.

Alternative or complementary approaches have been tested in the preclinical setup to improve graft survival. Autologous cell therapy products derived from host hiPSCs would theoretically be ideal from an immunological standpoint.^88^ Recent reports, however, suggest that mouse and human iPSC derivatives can be immunogenic in syngeneic or autologous recipients and in an autologous humanized mouse model, respectively.^89,90^ In addition, the current high cost of GMP-grade production of patient-specific hiPSCs renders therapeutic autologous hPSC-grafts unrealistic at this time. Other strategies have been described and partially tested to reduce or suppress human allogeneic immune responses against hPSC-derived cell therapy products. For example, encapsulation techniques, that are being tested on diabetic patients (NCT03163511: https://clinicaltrials.gov/ct2/show/NCT03163511?term=NCT03163511&draw=2&rank=1), can isolate implanted cells from the host but also preclude all cellular (including synaptic) interactions with it. Matching donor and host major histocompatibility complex (MHC) could be a way to avoid the immune system. Access to MHC matched donor lines can be ensured either via selection in the general population of HLA homozygous human induced PSC (iPSC) (e.g. A, B, DR triple homozygous) established by a global iPSC haplobank.^91^

One promising strategy is the generation of ‘universal cells’, also known as ‘hypoimmunogenic cells’ where hESC and hiPSCs can be engineered to reduce their immunogenicity upon transplantation, for example by use of CRISPR-Cas9 to disrupt HLA on their surface, while still maintaining their ability to be differentiated towards the neuronal type of interest. To achieve this goal and ensure safety, there is a need to optimize the engineering strategy.^92,93^ Results in partially MHC-matched allogeneic neural grafts in primates are controversial, showing increased survival in the short-term^94^ but no effect on rejection in the long-term.^32^ Transgenic expression of soluble immune-modulators by the cell therapy product^95^ or gene-editing approaches targeting non-polymorphic MHC-class I genes^96^ represent other avenues under investigation in ‘humanized’ mouse models.

There is currently no consensus as to which of these strategies can resolve the issue of allogeneic responses to hPSC neural grafts. Moreover, gene editing will introduce a raft of additional regulatory complications over and above those already confronting a stem-cell derived ATMP. In this light, despite the associated risks, chronic immunosuppression currently remains the best option to protect allogeneic grafts from rejection. Thus, immunology expertise must be utilized in planning transplant procedures in order to tailor induction and maintenance treatment to the individual, ensure long-term safety for the participant and long-term survival of the graft. The challenge of adherence to long-term immunosuppressant treatment is associated with that of finding the most appropriate readouts to monitor graft survival and immunogenicity triggered by grafted cells over time.

## Conclusion: bringing preclinical knowledge into a clinical setting

There are compelling reasons for considering regenerative medicine for the treatment of a wide range of neurodegenerative conditions, ranging from common heterogeneous conditions such as Parkinson’s disease to many rarer conditions, including single gene disorders such as Huntington’s disease. Together, these conditions represent a very large and growing disease burden, and the great majority are currently largely untreatable. Furthermore, for many conditions, targeted pharmacological treatments are a remote prospect as the detailed pathogenesis is not yet fully delineated, making a rational approach to therapy difficult or impossible. However, even where pathogenesis is obscure, a condition can still be amenable to cell therapy if the anatomy and distribution of neuronal or glial cell loss is characterized, in particular in conditions in which major cell loss affects relatively focal areas and/or predominantly involves a specific neural cell type.

As we move towards clinical trials for neural transplantation in neurodegenerative disease, it is essential that we incorporate and adapt understanding derived from preclinical studies, and that we recognize the complex, wide-ranging and multi-component challenges in evaluating delivery of substances and cells to the brain. We therefore propose the development of agreed upon research frameworks that are sufficiently flexible to accommodate the multiple complexities inherent in the development and evaluation process, and which will highlight future directions with the potential to expedite progress towards therapies for clinical benefit. We suggest that frameworks developed for Huntington’s disease will help to accelerate progress for a wide range of other neurodegenerative conditions.^17^

## Supplementary Material

awac086_Supplementary_Data

## Appendix 1

### SC4HD members

Further details are provided in the Supplementary material.

Anne-Catherine Bachoud-Levi (Paris Est University and Henri Mondor hospital, APHP, France), Gerhard Bauer (University of California, Davis, USA), Philipp Capetian (University Hospital Würzburg, Germany), Elena Cattaneo (University of Milan, Italy), Jeffersen Chen (University of California, Irvine, USA), Stephen Dunnett (Cardiff University, UK), Zdenka Ellederova (Institue of Animal Physiology and Genetics, Czech Republic), Mariah Lelos (Cardiff University, UK), Liangxue Lai (Guangzhou Institutes of Biomedicine and Health, China), Meng Li (Cardiff University, UK), Anna Morenkova (University of California, Irvine), Guangjin Pan (Guangzhou Institutes of Biomedicine and Health, China), Jack Reidling (University of California, Irvine, USA), Jiwhan Song (CHA University, South Korea), Pei Zhong (The First Affiliated Hospital, Sun Yat-Sen University, China).

## Abbreviations

ATMP =: advanced therapy medicinal product
EHDN =: European Huntington’s Disease Network
GPC =: glial progenitor cell
hESC =: human embryonic stem cell
hiPSC =: human induced pluripotent stem cell
hPSC =: human pluripotent stem cell
MHC =: major histocompatibility complex
MSN =: medium spiny neuron
NPCs =: foetal-derived neural progenitor cell
SC4HD =: Stem cells for Huntington’s disease
TPP =: target product profile
