# Supplementary material for: Translating cell therapies for neurodegenerative diseases: Huntington’s disease as a model disorder
Source: Brain. 2022 Mar 9;145(5):1584–97. doi: 10.1093/brain/awac086 (PMC9166564; doi:10.1093/brain/awac086)
Supplement: awac086_Supplementary_Data [file awac086_supplementary_data.pdf]

SC4HD members

Anne-Catherine Bachoud-Levi (Paris Est University and Henri Mondor hospital, APHP, France), Gerhard Bauer (University of California, Davis, USA), Philipp Capetian (University Hospital Würzburg, Germany), Elena Cattaneo (University of Milan, Italy), Jeffersen Chen (University of California, Irvine, USA), Stephen Dunnett (Cardiff University, UK), Zdenka Ellederova (Institue of Animal Physiology and Genetics, Czech Republic), Mariah Lelos (Cardiff University, UK), Liangxue Lai (Guangzhou Institutes of Biomedicine and Health, China), Meng Li (Cardiff University, UK), Anna Morenkova (University of California, Irvine), Guangjin Pan (Guangzhou Institutes of Biomedicine and Health, China), Jack Reidling (University of California, Irvine, USA), Jiwhan Song (CHA University, South Korea), Pei Zhong (The First Affiliated Hospital, Sun Yat-Sen University, China).
